# Assessing the readability and quality of online information on benign paroxysmal positional vertigo

**DOI:** 10.1308/rcsann.2022.0150

**Published:** 2023-02-07

**Authors:** H Raja, ZR Almansoor

**Affiliations:** ^1^ University Hospitals Birmingham NHS Foundation Trust, UK; ^2^ University of Manchester, UK

**Keywords:** BPPV, Otolaryngology, Consumer health information, Data accuracy, Reading, Comprehension, COVID-19

## Abstract

**Introduction:**

Benign paroxysmal positional vertigo (BPPV) is the most common cause of peripheral vertigo. It can have a significant impact on quality of life, with individuals often seeking information online for reassurance and education. The aim of this study is to assess the readability and quality of online information on BPPV.

**Methods:**

The terms ‘benign paroxysmal positional vertigo’ and ‘BPPV’ were entered into Google. The first 50 websites generated for each search term were screened. Readability was assessed using the Flesch–Kincaid Reading Ease Score (FRES), Flesch–Kincaid Grade Level (FKGL), Simple Measure of Gobbledygook (SMOG) Index and Gunning Fog Index (GFOG). Quality was assessed using the DISCERN instrument. Spearman’s correlation between quality and readability was calculated.

**Results:**

A total of 39 websites met the inclusion criteria. The mean and 95% confidence intervals for the FRES, FKGL, SMOG, GFOG and DISCERN scores were 50.2 (46.1–54.3), 10.6 (9.87–11.4), 10.1 (9.5–10.7), 13.6 (12.7–14.4) and 36.7 (34.6–38.7), respectively. Weak correlation was noted between DISCERN and FRES (*r_s_* = −0.23, *p* = 0.17).

**Conclusion:**

Online information on BPPV is generally of poor quality and low readability. It is essential that healthcare professionals inform their patients of this limitation and advocate for improved online patient education resources that are both high quality and easy to comprehend.

## Introduction

Benign paroxysmal positional vertigo (BPPV) is a common inner ear disorder that accounts for over half of all cases of peripheral vertigo. Lifetime prevalence is estimated to be 2.4%, with females two to three times more likely to be affected than males, and peak onset occurring in the fifth and sixth decades of life.^1,2^ BPPV occurs due to the displacement of otoconia within the semi-circular canals of the inner ear, commonly after positional changes of the head, with attacks typically lasting less than a minute.^3^ Vertiginous attacks can range in severity, from mild to debilitating episodes that may induce nausea or vomiting, and significantly hinder quality of life.^3,4^

Although repositioning techniques such as the Epley manoeuvre can often be successful in managing BPPV, recurrence of attacks is frequent, with around 70% of patients having a further attack within the first year.^5^ This can have profound personal, social and financial implications for both patients and caregivers. Improved patient understanding of BPPV is therefore important to help avoid unnecessary delays in diagnosis and treatment, as well as to better manage expectations.^1^

The growing rise in technology and, more recently, social media has led to patients resorting increasingly to the internet for their medical information.^6^ This has been amplified further following the COVID-19 pandemic, with an increasing shift towards online consultations and a reduction in face-to-face appointments with healthcare professionals.^7,8^ The internet, however, is largely unregulated and so online information may be inaccurate and difficult to read.^9^ Owing to the high prevalence of BPPV and the potential for recurrent attacks,^5^ this is something that patients can ill afford.

To date, no study has examined the appropriateness of online information on BPPV. This study aimed to assess the readability and quality of online information on BPPV.

## Methods

### Internet search methods

**Figure 1:**
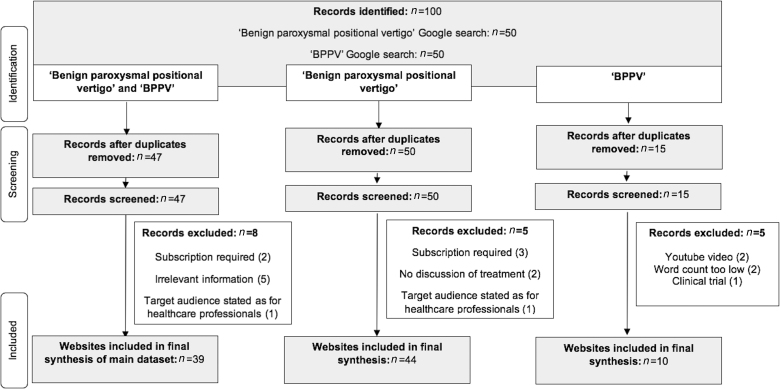
Flow diagram displaying the systematic search methodology. The searches were performed in March 2022.

Online information using the search terms ‘benign paroxysmal positional vertigo’ and ‘BPPV’ was assessed separately in March 2022 using Google. Cookies and browser history were deleted before the search to avoid the effects of previous internet use on search results. The first 50 webpages for each search term were assessed for their readability and quality, with Google being used as the main search engine for our study owing to it occupying the vast majority of the market share.^10^ The workflow of our methodology is shown in Figure 1.

All the websites were assessed objectively by two independent assessors (H.R. and Z.R.A.). If there was an inconsistency regarding the evaluation of a website, then a third independent assessor evaluated the website and made the final decision.

### Eligibility criteria

Websites that were only accessible and written in English were included. If websites provided irrelevant information or did not discuss treatment for BPPV, they were excluded. Other exclusion criteria included: if target audience was healthcare professionals, the word count was below the lower limit for readability scoring and duplicates.

### Readability assessment

Readability was assessed using four validated scoring tools: the Flesch–Kincaid Grade Level (FKGL), Flesch Reading Ease Score (FRES), Gunning Fog Index (GFOG) and Simple Measure of Gobbledygook (SMOG) score (Table 1). Written information from each of the websites was scored using an online scoring tool.^13^ The scoring for each tool and how scores correspond to reading difficulty is shown in Table 2.

**Table 1:** Formulae for readability tools

Readability tool	Formula
FKGL	0.39 × (average number of words per sentence) + (11.8 × average number of syllables per word) – 15.59
FRES	206.835 – (1.015 × number of words/ number of sentences) – (84.6 × number of syllables/ number of words)
GFOG	0.4 × [words/sentences) + 100 (complex words/total words)]
SMOG	√number of polysyllabic words + 3

FKGL = Flesch–Kincaid Reading Ease Score; FRES = Flesch–Kincaid Grade Level; GFOG = Gunning Fog Index; SMOG = Simple Measure of Gobbledygook

Adapted from Wang *et al*^11^ and O’Connell Ferster and Hu^12^

**Table 2:** Scoring of readability tools and their corresponding difficulty

Readability tool scoring				Reading difficulty
FRES	FKGL	SMOG	GFOG	
0–30	College graduate	17+	17+	Very difficult
31–50	College level	13–16	13–16	Difficult
51–60	High school	9–12	10–12	Fairly difficult
61–70	Eighth–ninth grade	8	8–9	Standard
71–80	Seventh grade	7	7	Fairly easy
81–90	Sixth grade	6	6	Easy
91–100	Fifth grade	5	5	Very easy

FKGL = Flesch–Kincaid Reading Ease Score; FRES = Flesch–Kincaid Grade Level; GFOG = Gunning Fog Index; SMOG = Simple Measure of Gobbledygook

Adapted from O’Connell Ferster and Hu^12^ and Boztas *et al*^14^

### Quality assessment

Quality of online information was assessed using the DISCERN tool. This validated scoring tool consists of 15 questions designed to evaluate both the reliability and quality of information using a 5-point Likert scale. An additional 16th question consisting of an overall rating is also included, with a score of 1 representing serious and extensive omissions of information, whereas a score 5 means that there are only minor and minimal omissions.^15^ Total DISCERN scores can be interpreted as the following: very poor (16–29), poor (30–40), fair (41–51), good (52–63) and excellent (>64).

### Correlation assessment

Spearman’s correlation was used to determine whether there was a trend between quality (DISCERN scores) and readability (FRES scores).

### Statistical analysis

Statistical analyses were performed using Microsoft Excel® 2019. Statistical significance was set at *p *< 0.05.

## Results

In total, 100 websites were screened against the exclusion criteria. This resulted in 39 unique websites constituting the main data set (Figure 1). The reasons for exclusion of 61 websites were as follows: duplicate websites (*n* = 53), requiring a subscription (*n* = 2), websites containing information irrelevant to BPPV (*n* = 5) and healthcare professionals being the target audience (*n* = 1).

### Readability assessment

Mean readability scores and 95% confidence intervals (CI) for FRES, FKGL, SMOG and GFOG were 50.2 (46.1–54.3), 10.6 (9.87–11.4), 10.1 (9.5–10.7) and 13.6 (12.7–14.4), respectively (Table 3). These scores are equivalent to the reading age of a 15–18 year old, 15–16 year old, 11–12 year old and 15–17 year old, respectively.

**Table 3:** Summary of readability and quality data for the terms ‘benign paroxysmal positional vertigo’ and ‘BPPV’

Test	Main data set search term analysis	Search term-specific subgroup analysis		
	‘Benign paroxysmal positional vertigo’ and ‘BPPV’ (*n* = 39)	‘Benign paroxysmal positional vertigo’ (*n* = 44)	‘BPPV’ (*n* = 10)	*p*-value
Flesch Reading Ease Score mean (95% CI)	50.2 (46.1–54.3)	50.6 (46.8–54.4)	43.2 (39.8–46.6)	0.167
Flesch–Kincaid Grade Level mean (95% CI)	10.6 (9.87–11.4)	12.1 (11.4–12.8)	10.3 (8.5–12.2)	0.084
Simple Measure of Gobbledygook mean (95% CI)	10.1 (9.5–10.7)	10.3 (9.2–18.2)	9.5 (8.2–10.8)	0.276
Gunning Fog Index mean (95% CI)	13.6 (12.7–14.4)	15.3 (14.5–16.1)	12.8 (10.8–14.8)	0.084
DISCERN score mean (95% CI)	36.7 (34.6–38.7)	37.9 (35.6–40.2)	43.7 (35.6–40.2)	0.026

BPPV = benign paroxysmal positional vertigo; CI = confidence interval

*p *< 0.05 for significance.

On sub-analysis of the search term ‘benign paroxysmal positional vertigo,’ the 44 websites had an average score and 95% CI of 12.1 (11.4–12.8), 50.6 (46.8–54.4), 15.3 (14.5–16.1) and 10.3 (9.2–18.2) for FKGL, FRES, GFOG and SMOG, respectively. These scores are equivalent to the reading age of a 15–18 year old, 21 year old, 15–17 year old and 12–13 year old, respectively.

On sub-analysis of the search term ‘BPPV,’ the ten websites had average scores and 95% CI of 10.3 (8.5–12.2), 43.2 (39.8–46.6), 12.8 (10.8–14.8) and 9.5 (8.2–10.8) for FKGL, FRES, GFOG and SMOG, respectively These scores are equivalent to the reading age of an 18–21 year old, 21 year old, 17–18 year old and an 11–12 year old, respectively.

### Quality assessment

The average DISCERN score and 95% CI when using the main data set was 36.7 (34.6–38.7). Only one website had a DISCERN score of good, with none having a score of excellent. Some 78.9% of websites had poor/very poor reliability and 59% had poor/very poor quality. All websites had potentially important but not serious deficiencies.

With regards to quality and reliability, the average DISCERN score and 95% CI across the websites using the search term ‘benign paroxysmal positional vertigo’ was 37.9 (35.6–40.2). Some 57% of websites had poor/very poor quality information, with 77% of websites having poor/very poor reliability. All websites had potentially important but not serious deficiencies.

When using the search term ‘BPPV’, the average DISCERN score and 95% CI for quality and reliability was 43.7 (35.6–40.2). This means that the websites were overall fair in terms of quality, with 20% of the websites having a DISCERN score of good, and none scoring as excellent. Some 70% of the websites were of poor/very poor reliability with 30% being of poor/very poor quality. In terms of quality, two websites had a quality deemed as excellent. Three websites were found to have a poor quality of information.

### Correlation between FRES and DISCERN

**Figure 2:**
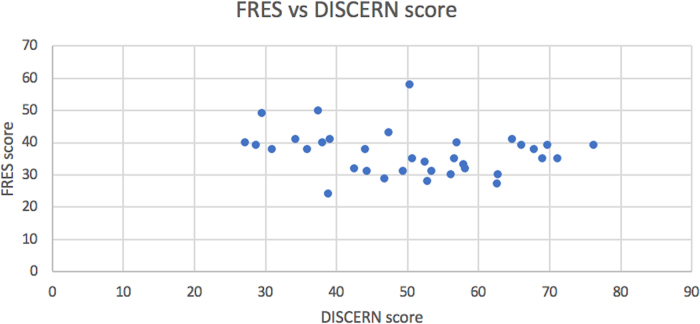
Scatter graph of Flesch Reading Ease Score (FRES) against DISCERN score for the main data set

There was a weak negative correlation between the FRES and DISCERN scores (*r_s_* = −0.22599, *p* = 0.17; Figure 2).

## Discussion

The internet is a growing and significant source of healthcare information for patients. It can aid communication, empower patients with decision making and improve the patient–doctor relationship. Caution should be exercised, however, because the internet can be largely unregulated, with potential for misinformation to surface that can lead to adverse health outcomes. To our knowledge, this is the first study to have comprehensively evaluated the readability and quality of websites providing information on BPPV for patients. Online information on BPPV was generally found to be of poor quality and written at a reading difficulty beyond what the average person would be able to understand.

Our readability data showed that a significant proportion of websites have a reading level greatly beyond the recommendation of 11 years.^8^ Given that the average reading age in the United Kingdom (UK) is between 9 and 15 years old,^16,17^ we found that many websites are written beyond what the average patient is capable of understanding. The 39 websites from the main data set were shown to have a high reading level in line with that of a 15–16 year old or higher, ranging from fairly difficult to read to very difficult to read. However, these findings contradict those obtained from SMOG scoring, which suggested that a significant proportion of the websites had a reading level of an 11–12 year old. This discrepancy may be explained by the fact that SMOG is calculated on the principle that the frequency of polysyllabic words within a sentence correlates with the reading difficulty of the text, even though not all polysyllabic words are difficult to understand.^18^ In the context of BPPV, this discrepancy suggests that the polysyllable count may not be an effective discriminator of reading difficulty. Overall, the fact that three of the four readability scores were consistent with the websites having a high reading level is strong evidence in favour of this being the case. This is further enforced by the fact that individually none of the websites were at the recommended reading level for FKGL and GFOG scoring, with only one website individually being at the recommended reading level based on FRES scoring.^19^

When comparing trends for the individual search terms, the search term ‘BPPV,’ resulted in websites having lower average scores for FRES and FKGL, meaning that they were more difficult to read than websites for the search term ‘benign paroxysmal positional vertigo’. However, ‘BPPV’ also resulted in the websites having a higher average GFOG and SMOG score, which correlates with an easier reading level. This suggests that using the full search term or abbreviated search term makes no difference to the reading difficulty across websites. It is of note, however, that because 35 duplicates were removed when conducting the search term ‘BPPV’ the impact that they would have had on the reading difficulty if they had been included is unknown.

Overall, these findings about readability raise major concerns with regards to the extent that patients are able to understand and interpret information on BPPV. This is problematic because it can lead to delays in patients receiving medical support and appropriate treatment for BPPV. As a result, patients may have an increased likelihood of experiencing adverse events, such as falls, and a lower quality of life. These concerns, however, are not only limited to BPPV, with the issue of reading difficulty of online material also being apparent in other conditions within otorhinolaryngology.^20,21^

Improving the readability of online material on BPPV could take the form of implementing more pictograms, diagrams and images to accommodate individuals who have a reading ability below that of a fifth grader.^22,23^ Other approaches could include greater spacing of the text and a change of font in terms of style and size.^22^ We also suggest direct involvement of patients in the writing of health information of BPPV to further enhance readability.^20^ However, in an attempt to avoid patronising the patient with simplified information and to ensure that those with higher reading abilities obtain sufficient information and understanding, it would be advisable to provide more complex sources of information alongside the basic information provided on websites. Additionally, we suggest that authors test the readability level of their content prior to publication, using readability tools such as FRES and FKGL scoring, although not SMOG, to ensure that their content is set at the target readability level of 11 years of age.

In terms of the quality of online information relating to BPPV, we found that it was generally poor. This was mainly due to websites providing sparse information or lacking information on how treatment will affect patients’ quality of life and a discussion of treatment options. The category that scored the lowest when assessing the reliability of information was the clarity of aims. This was followed by referral to areas of uncertainty. The two categories that scored the lowest when assessing information quality were provision of support for shared decision making and describing risks for each treatment. These areas should, therefore, be prioritised to improve the quality of online information relating to BPPV. This is important because it will facilitate better doctor–patient decision making, leading to improved treatment adherence and patient outcomes, and ensuring that patients are aware of which information is evidence-based.

When analysing DISCERN scoring for the separate search terms, ‘BPPV’ was shown to be of fair quality, whereas ‘benign paroxysmal positional vertigo’ was shown to be of poor quality overall. In terms of reliability, ‘BPPV’ scored higher than ‘benign paroxysmal positional vertigo’, with a score of 48.5% compared with 46%, although both were classed as fair. In terms of quality, ‘BPPV’ scored significantly higher than ‘benign paroxysmal positional vertigo’ at 60.3% compared with 48%. This translates to fair and poor quality, respectively. The categories that scored the lowest in terms of reliability were clarity of the aims and referral to areas of uncertainty for both search terms. The categories that scored the lowest in terms of quality were providing support for shared decision making and how treatment affects the quality of life for both. This demonstrates that information obtained using the search term ‘BPPV’ yields more reliable, high-quality information than the search term ‘benign paroxysmal positional vertigo’.

Weak correlation was found between the readability and quality of online material for BPPV. This is problematic because, although high-quality websites that are hard to read would pose little difficulty for highly educated patients, the layperson will struggle to fully comprehend the information provided. Similarly, easily readable content that lacks quality can misinform patients and adversely affect health outcomes. Although further work is needed to improve online information on BPPV, we recommend that patients are directed to use Association of Chartered Physiotherapists Interested in Vestibular Rehabilitation and Healthline websites because they were best in terms of both quality and readability.^24,25^

Although benign and often self-limiting, BPPV can have a considerable impact on quality of life. It is, therefore, important to improve health literacy of these patients, defined as the ability to understand and use information to appropriately manage health, by directing them towards or creating easily readable and high-quality online material. Importantly, we suggest that healthcare professionals become more heavily involved in the production of online information for BPPV. This will ensure that important information is not omitted and content is accurate and comprehensive. We also recommend that there is increased patient involvement in the production of online content to ensure that information is easy to read, understandable and based on patients’ needs. To ensure patients are using accurate and reliable sources of information that are in line with their reading ability, we also propose that all healthcare websites on BBPV provide information about the reading age, reliability and quality of their content, so that patients can make an informed choice about which websites are most suitable to use.

### Limitations

Our study had several limitations. First, readability tools fail to assess audio-, image- or video-based information, including the presentation of information online. These are important factors that aid comprehension of online material.^26^ We may have, therefore, underestimated the readability of websites. Second, scoring quality of information using the DISCERN tool requires subjective evaluation, which somewhat reduces the reproducibility of our results. Third, Google was the only search engine used to identify information on BPPV. It is likely that we may not have produced findings that are truly representative of the patient experience as other search engines, such as Bing or Yahoo, could have resulted in different findings. Finally, the internet is a fluid, ever-changing system so our search results may differ in the future.

## Conclusion

This is the first study to have systematically evaluated the quality and readability of online information for BPPV. We found that websites about BPPV were generally of poor quality and written at a reading level above the recommended reading age. A weak correlation was noted between readability and quality of online material for BPPV. Healthcare professionals should be aware of these limitations and advocate for improved online patient education resources that are both high quality and easy to comprehend.

## Author contributions

H.R. conceived the idea of writing the manuscript. H.R. and Z.R.A. equally designed the methodology, acquired and analysed the data, and drafted the manuscript. H.R. approved the manuscript for submission. H.R. agrees to be accountable for all aspects of the work.
